# Characterization of a semidominant dwarfing *PROCERA* allele identified in a screen for CRISPR/Cas9‐induced suppressors of loss‐of‐function alleles

**DOI:** 10.1111/pbi.13027

**Published:** 2018-11-09

**Authors:** Zhiguo Zhu, Xiaojun Kang, Vai S. Lor, David Weiss, Neil Olszewski

**Affiliations:** ^1^ Department of Plant and Microbial Biology and the Microbial and Plant Genomics Institute University of Minnesota St. Paul MN USA; ^2^ Institute of Plant Sciences and Genetics in Agriculture The Robert H. Smith Faculty of Agriculture, Food and Environment The Hebrew University of Jerusalem Rehovot Israel; ^3^ Present address: Laboratory of Molecular Biology of Tomato Bioengineering College Chongqing University Chongqing China; ^4^ Present address: Department of Agronomy University of Wisconsin 1575 Linden Drive Madison WI 53706 USA

**Keywords:** mutagenesis, CRISPR/cas9, gain‐of‐function mutant, PROCERA, dwarf, reduced GA sensitivity, semidominant, seed germination

DELLA proteins were first identified as inhibitors of gibberellin (GA) signalling but have since been shown to be involved in regulation of many growth responses. GA causes responses by triggering the destruction of DELLA proteins (Willige *et al*., 2007). These proteins contain a conserved DELLA domain, which is followed by a GRAS domain. The GRAS domain which defines a family of proteins, including the DELLA proteins, is a conserved domain named after the first three family members: GAI (gibberellic‐acid insensitive), RGA (Repressor of *ga1‐3*), and SCR (Scarecrow). The DELLA domain encompasses the DELLA, LExLE and TVHYNP motifs. These motifs are important for binding to the GA‐bound GA receptor GID1, which is one of the initial steps in targeting the DELLA proteins for destruction by the 26S proteasome (Dill *et al*., 2001). The GA‐GID1‐DELLA complex interacts with the SLY1/GID2 SCF complex, which marks it for destruction by modifying it with ubiquitin. In this way, the suppression of GA signalling by DELLA is released and GA responses are activated. A number of mutants with mutations affecting the DELLA domain that increase the abundance of DELLA by weakening the interaction with the GA‐GID1 complex are known (Ueguchi‐Tanaka *et al*., 2007; Willige *et al*., 2007). Since these mutant DELLA proteins retain the ability to inhibit GA responses, the mutants are dwarfed.

Tomato has one DELLA protein called PROCERA (PRO). There is a well‐studied partial loss‐of‐function mutant, *pro*, which has a single amino acid substitution, valine to glutamate, in the VHVID motif of its GRAS domain (Bassel *et al*., 2008; Jupe *et al*., 1988). Strong recessive *pro* loss‐of‐function alleles, *pro* ^*TALEN*^ and *pro* ^*∆GRAS*^, were generated using transcription activator‐like effector nucleases (TALENs) and an Ac/Ds system, respectively (Livne *et al*., 2015; Lor *et al*., 2014). Consistent with these mutations fully activating GA signalling, homozygous mutants are extremely tall, have light green leaves with smoother leaf margins, and do not respond to the GA biosynthesis inhibitor paclobutrazol (PAC) or GA treatment. Recently, a gain‐of‐function allele affected in the DELLA motif that causes mild dwarfing and reduced GA sensitivity was reported (Tomlinson *et al*., 2018) but strong gain‐of‐function alleles have not been reported. Here, we report the generation and characterization of strong dwarfing alleles produced using the clustered regularly interspaced short palindromic repeats (CRISPR)/CRISPR‐associated protein9 (Cas9) system to induce intragenic suppressor mutations of *pro* ^*TALEN*^ alleles.

In the course of experiments to identify regenerating shoots in which the *pro* ^*TALEN*^ loss‐of‐function mutation has been repaired due to CRISPR/cas9 stimulated homologous recombination, we recovered several dwarf plants with dark green serrated leaves (Figure 1a, b). The regenerated T_0_ plants were dwarfs and the dwarfing alleles *PRO* ^*GF8*^ and *PRO* ^*GF9*^ from plants #8 and #9, respectively, were heritable. Sequencing confirmed that the *PRO* ^*GF8*^ and *PRO* ^*GF9*^ are intragenic suppressors of *pro* ^*TALEN1*^ and *pro* ^*TALEN7*^ respectively and that each encodes a full‐length mutant protein (Figure 1c, d). The protein encoded by *PRO* ^*GF8*^ is predicted to have a 12 amino acid insertion and 2 amino acid substitution affecting the LExLE motif, while the protein encoded by *PRO* ^*GF9*^ has a three amino acid deletion and an E to R substitution that affects the LExLE motif. *PRO* ^*GF8*^ was characterized further. F_1_ seedlings from a cross between plant #8 and wild‐type (M82) were either extreme dwarfs with dark green leaves or wild‐type in appearance (Figure 1e). Only the dwarf plants had inherited the *PRO* ^*GF8*^ allele indicating that it was dominant or semidominant.

**Figure 1:**
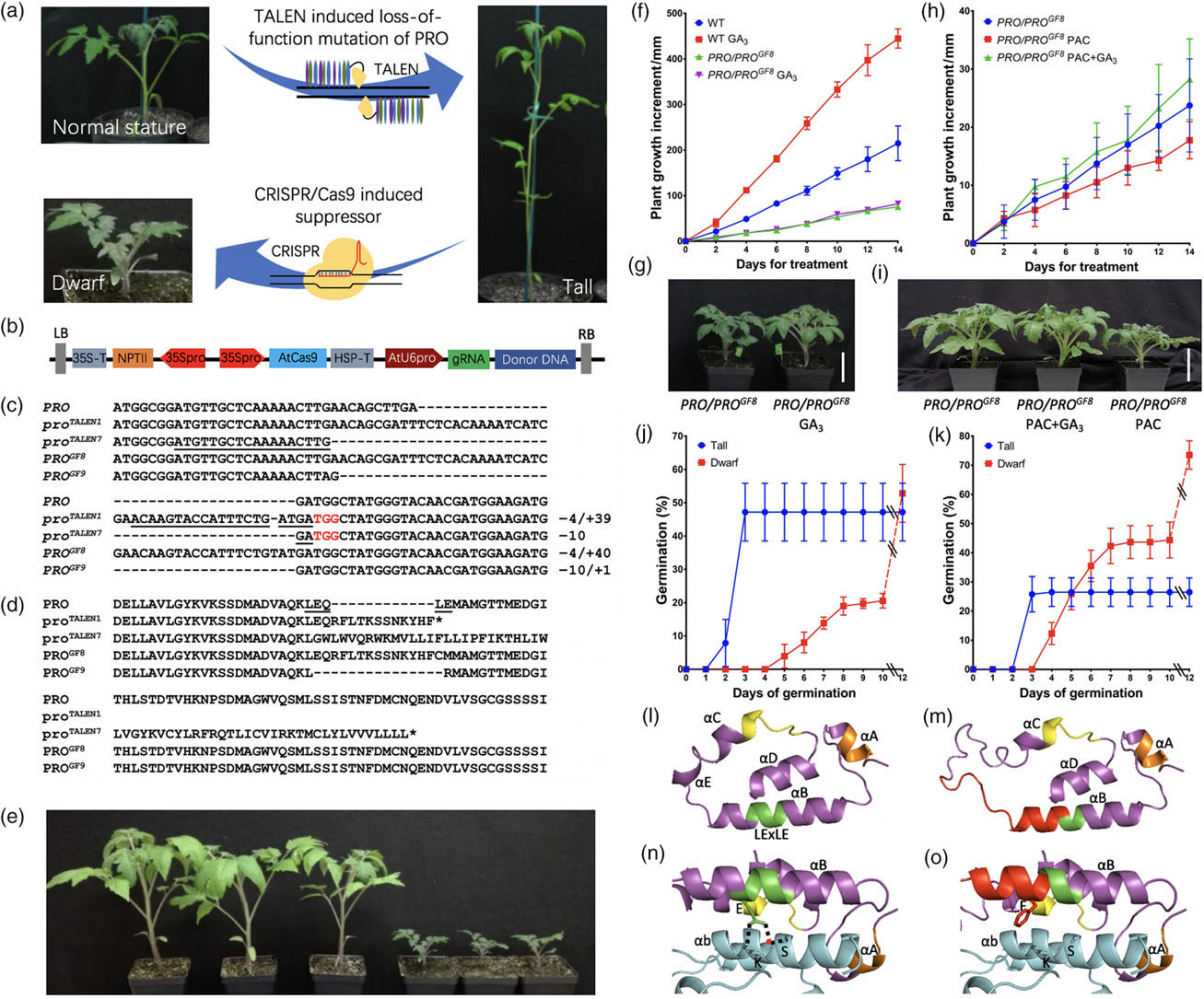
(a) Strategy for generating *pro* ^*TALEN*^ loss‐of‐function alleles and subsequent generation of suppressor mutations. (b) T‐DNA construct used to edit *pro* ^*TALEN*^ mutation sites were constructed in pTRANS220 as described by previously (Čermák *et al*., 2017). The gRNAs targeting *pro* ^*TALEN*^ ^*1*^ and *pro* ^*TALEN*^ ^*7*^ were constructed in pMOD_B2515 and the resulting module (B) was assembled together with modules from pMOD_A0101 (module A), and pMOD_C0000 (module C). Module C consisted of a fragment of genomic DNA spanning the *pro* ^*TALEN*^ mutation site. (c) DNA and (d) protein sequence of CRISPR/Cas9 induced alleles. The gRNA binding site is underlined and PAM site is highlighted in red in the *pro* ^*TALEN*^ ^*1*^ and *pro* ^*TALEN*^ ^*7*^ sequences. The size of insertion (+) and deletion (−) compared with wild‐type is indicated to the right of sequences. The conserved LExLE motif of DELLA protein is underlined in the wild‐type sequence. (e) Four‐week old F_1_ seedlings from a cross between T_0_ plant #8 and M82. Left to right, three tall plants that lack *PRO* ^*GF*^ ^*8*^ and three *PRO/* *PRO* ^*GF*^ ^*8*^ plants. (f, h) Plants were spayed to runoff every other day with 0.07% ethanol (control treatment), 50 μm GA_3,_ 100 μm Paclobutrazol (PAC) or GA_3_ plus PAC and the height was measured every other day for 2 weeks. After 14 days of treatment, the height of control and GA_3_‐treated *PRO/* *PRO* ^*GF*^ ^*8*^ plants were similar. After 14 days, PAC treated *PRO/* *PRO* ^*GF*^ ^*8*^ were significantly shorter than GA_3_‐treated *PRO/* *PRO* ^*GF*^ ^*8*^ plants based on an ANOVA followed by *t*‐test (*P* value 0.0014). (g) Seven‐week old *PRO/* *PRO* ^*GF*^ ^*8*^ plants after 2 weeks of treatment as in panel f. (i) Seven‐week old *PRO/* *PRO* ^*GF*^ ^*8*^ plants after 2 weeks of treatment as in panel h. Scale bar, 50 mm. (j) and (k), Time course of germination of seeds giving rise to tall and dwarf seedlings from (j) 4 month‐old F_1_ seeds from crossing T_0_ plant #8 with wild‐type pollen and (k) seeds immediately after harvest from a selfed *PRO/pro* ^*GF*^ ^*8*^ plant. Seeds that had not germinated after 10 days were scarified by removing the endosperm and seed coat adjacent to the root tip. Dash lines indicate the germination after scarification, which was scored at day 12. At least 40 seeds are used for each test, which was repeated three times. (l‐o) 3D models of PROCERA (l) and PRO^GF^ ^8^ (m) based on template 2ZSH (GA_3_‐GID1A‐GAI). Close‐up view of the hydrogen bonds between LExLE motif of PROCERA and αb of GID1 (n) LExLE motif of proGF8 and αb of GID1 (o) based on template. The DELLA motif is highlighted in orange and TVHYNP motif in yellow. The LExLE motif in PRO and PRO^GF^ ^8^ is highlighted in green; the insertion of PRO^GF^ ^8^ is highlighted in red. Hydrogen bonds are indicated as dot lines. A water molecule bridging E and S is showed as a red sphere.

We tested the effect of *PRO* ^*GF8*^ on GA responsiveness. In contrast to wild‐type M82 plants, which grew more rapidly when sprayed every 2 days with 50 μM GA_3_, *PRO/PRO* ^*GF8*^ were unaffected (Figure 1f, g). Because DELLA proteins promote GA synthesis, endogenous GA levels are highly elevated in plants carrying *DELLA* gain‐of‐function alleles (Talón *et al*., 1990). To determine if the apparent GA insensitivity of *PRO/PRO* ^*GF8*^ plants is because they contain saturating levels of endogenous bioactive GA, we treated *PRO/PRO* ^*GF8*^ (Figure 1h, i) and wild‐type (not shown) plants with the GA biosynthesis inhibitor paclobutrazol (PAC) or a combination of PAC and GA_3_. PAC treatment reduced the height of both *PRO/PRO* ^*GF8*^ and wild‐type plants and the GA_3_ treatment reversed this effect. The effects of the treatments were much smaller for *PRO/PRO* ^*GF8*^, indicating that, while *PRO/PRO* ^*GF8*^ responds to GA_,_ it is nearly insensitive to it.

While germination tests found that initially only one half of the newly harvested F_1_ seeds from a cross between the T_0_ plant #8 and M82 germinated, the non‐germinated seeds germinated after they were scarified. The seedlings from seeds that did not require scarification were tall and did not carry the *PRO* ^*GF*^ allele while all of the seedlings from seeds that required scarification were *PRO/PRO* ^*GF8*^ and dwarf (not shown). When we examined the germination kinetics after 4 months of storage, seeds that germinated by day 3 gave rise to tall plants whereas all seeds that germinated after day 3 produced dwarf seedlings (Figure 1j). After 10 days, 30% of the seeds had not germinated. Following scarification, these ungerminated seeds germinated and gave rise to dwarf seedlings.

We also tested germination of fresh seeds harvested from selfed *PRO/PRO* ^*GF8*^ (Figure 1k). After 3 days, all of the seeds that produced wild‐type stature seedlings had germinated (26%). The seeds that had not germinated could be divided into two groups. One group comprising 44% of the seeds germinated by day 10 and produced dwarf seedlings. The remaining seed required scarification and also produced dwarf seedlings. The observed ratios for the tall seedlings, dwarf seedlings from seed that germinated without scarification, and dwarf from seeds requiring scarification [Tall vs. Dwarf (non‐scarified) vs. Dwarf (scarified)] fit a 1 : 2 : 1 ratio (three trials Chi‐square range from 0.458 to 3.471, *P* value from 0.176 to 0.795), which suggested that the *PRO* ^*GF8*^ allele is semidominant. Consistent with this hypothesis, when genotyped, 16 out of 16 dwarf plants from seeds that germinated without scarification were heterozygous and 10 of 11 dwarf seedlings from seeds requiring scarification were homozygous for *PRO* ^*GF8*^ and the remaining plant was heterozygous. Plants from seeds that require scarification to germinate were shorter than the dwarf plants from seeds that germinated without scarification (not shown) indicating that *pro* ^*GF8*^ is also semidominant with respect to plant stature.

Molecular modelling of PRO and PRO^GF8^ predicts that PRO^GF8^ protein has a longer disordered region between the LExLE and TVHYNP motifs and the intervening alpha helix structure(s) are also affected (Figure 1l–o). The changes in PRO^GF8^ are predicted to weaken its interaction with the GA‐bound GID1 because the stabilizing interaction between the second glutamic acid in LExLE motif and arginine and lysine in GID1 are disrupted. Weakening the interaction with GID1 likely increases the abundance of PRO^GF8^.

## Conflict of interest

The authors declare that there is no conflict of interest.
